# Take a load off: examining partial and complete cognitive offloading of medication information

**DOI:** 10.1186/s41235-023-00468-z

**Published:** 2023-02-08

**Authors:** Lauren L. Richmond, Julia Kearley, Shawn T. Schwartz, Mary B. Hargis

**Affiliations:** 1 Department of Psychology, Stony Brook University, Psychology B Building, Stony Brook, NY 11794 USA; 2 Department of Psychology, McGill University, Montreal, Canada; 3 Department of Psychology, Stanford University, Stanford, USA; 4 Department of Psychology, Texas Christian University, Fort Worth, USA

**Keywords:** Cognitive offloading, Value-based remembering, Medication information, Partial offloading

## Abstract

Although cognitive offloading, or the use of physical action to reduce internal cognitive demands, is a commonly used strategy in everyday life, relatively little is known about the conditions that encourage offloading and the memorial consequences of different offloading strategies for performance. Much of the extant work in this domain has focused on laboratory-based tasks consisting of word lists, letter strings, or numerical stimuli and thus makes little contact with real-world scenarios under which engaging in cognitive offloading might be likely. Accordingly, the current work examines offloading choice behavior and potential benefits afforded by offloading health-related information. Experiment 1 tests for internal memory performance for different pieces of missing medication interaction information. Experiment 2 tests internal memory and offloading under full offloading and partial offloading instructions for interaction outcomes that are relatively low severity (e.g., sweating). Experiment 3 extends Experiment 2 by testing offloading behavior and benefit in low-severity, medium-severity (e.g., backache), and high-severity interaction outcomes (e.g., heart attack). Here, we aimed to elucidate the potential benefits afforded by partial offloading and to examine whether there appears to be a preference for choosing to offload (i) difficult-to-remember information across outcomes that vary in severity, as well as (ii) information from more severe interaction outcomes. Results suggest that partial offloading benefits performance compared to relying on internal memory alone, but full offloading is more beneficial to performance than partial offloading.

## Introduction

Recently, there has been much interest in developing and understanding memory improvement strategies, from cognitive training regimes (see for example Chein & Morrison, 2010; Jaeggi et al., 2008; Redick et al., 2013; Richmond et al., 2014; von Bastian et al., 2013; Wiemers et al., 2018) to strategy training (see Hudes et al., 2019 for a recent overview). One simple strategy that people often use to reduce the burden on internal memory in their everyday lives involves creating external reminders—for example, creating a digital calendar entry to remind oneself of a future doctor’s appointment. This strategy, in which externalizing information serves to reduce demands on internal memory, has been dubbed “cognitive offloading” (Risko & Gilbert, 2016). Although cognitive offloading is common in everyday life, relatively little is known about (i) the conditions under which it can benefit memory, (ii) the internal memory consequences of offloading for non-offloaded information, and (iii) how decisions are made about what should be offloaded.

Alongside the emerging scientific interest in cognitive offloading, a large body of research on the effect of cues on memory should be considered, given that cognitive offloading often involves generating and using cues intended to serve as reminders for important information. Beginning with Slamecka’s (1968) seminal work examining the consequences of providing parts of previously studied lists as cues at test (i.e., the part-set cuing paradigm), the provision of cues at test has been demonstrated to result in *poorer* memory performance compared to a free recall condition in which no cues were provided. This performance decrement in part-set cuing has been tied to the retrieval disruption hypothesis, suggesting that the provision of cues serves to disrupt an individual's own idiosyncratic retrieval organization (Basden & Basden, 1995). Instead, when participants are left to their own devices (i.e., when cues are not provided), participants are free to organize recall in any fashion they wish, thereby avoiding the disruptive impact of provided cues. However, the difference between predicted performance and actual performance has been shown to be larger for participants in the part-set cuing condition compared to the control-free recall condition (Rhodes & Castel, 2008). The gap observed between predicted and actual performance in the presence of cues suggests that participants are not well-calibrated to the costs associated with part-set cuing, and this metacognitive miscalibration may induce learners to rely on external cues more than is optimal (see Sachdeva & Gilbert, 2020).

On the other hand, there are some circumstances under which the presence of cues can be helpful to memory performance. For example, age differences in performance have been shown to be eliminated when participants are provided with category names as cues at test (Smith, 1977), which contrasts the typical age-related differences observed on free recall tests (i.e., that young adults outperform older adults; see for example Perlmutter, 1979). Moreover, cues can bring to mind previously inaccessible information, such as by supplying a participant with a word and telling them that the supplied word rhymes with the target word in a paired-associates task (Tulving, 1974). Cues can also serve as a reminder for information that was not directly contained in the cue (Ratcliff & McKoon, 1994). The fact that cues can both help and hinder memory performance is interesting from the standpoint of cognitive offloading, in which participants create and use external reminders—essentially, *memorial cues*—to improve the performance of memory-based activities in everyday life.

To illustrate the varying consequences of cues on memory performance, imagine a shopper in a grocery store who has prepared a grocery list. During the shopping trip, the shopper may consult the list to help them successfully select and purchase the items on their list. It is also possible that, in this context, the shopper may have a more difficult time remembering to pick up any last-minute item(s) that did not make it onto the list. If, however, some items on the list cue retrieval for other, non-listed items, such as the list item “peanut butter” cuing the shopper to also remember to purchase jelly, then this shopper may be able to successfully leave the grocery store with all necessary items.

In the laboratory, cognitive offloading has been shown to improve performance on memory-based tasks relative to relying solely on internal memory for both retrospective (e.g., Kelly & Risko, 2019; Morrison & Richmond, 2020; Risko & Dunn, 2015) and prospective memory (e.g., Boldt & Gilbert, 2019; Sachdeva & Gilbert, 2020) information. These effects have also been observed using a variety of different study materials, including letters (Morrison & Richmond, 2020; Risko & Dunn, 2015), numbers (Boldt & Gilbert, 2019; Sachdeva & Gilbert, 2020), and word lists (Kelly & Risko, 2019). Importantly, cognitive offloading has also been shown to result in costs to internal memory when the external store is unexpectedly unavailable (Kelly & Risko, 2019), and higher rates of false recall have been observed when the external store is manipulated compared to relying solely on internal memory (Lu et al., 2020; Risko et al., 2019).

One interesting facet of this research has focused on participants’ choices about offloading to the external environment. Participants choose to offload more when under higher memory loads (Gilbert, 2015; Morrison & Richmond, 2020; Risko & Dunn, 2015). However, choice behavior does not always follow such sensible patterns. Participants with weaker internal memory capacities surprisingly do not appear to offload more items than those with stronger internal memory abilities (Morrison & Richmond, 2020; but see Risko & Dunn, 2015) even though it would be advantageous for them to do so. Similarly, Boldt and Gilbert (2019) have shown that participants’ metacognitive beliefs about their memories have a larger impact on offloading choice than objective memory performance does: Participants were generally underconfident in their memory abilities and engaged in more offloading than necessary given their internal memory abilities (Boldt & Gilbert, 2019; Scarampi & Gilbert, 2021). That is, participants can exhibit overreliance on offloading mechanisms, even to a fault. Interestingly, this effect is reversed in older adults, such that older participants exhibit metamemory overconfidence and offload *less* often than would be optimal (Scarampi & Gilbert, 2021). Together, these findings serve to demonstrate that offloading choice behavior is not always well-tied to internal memory ability.

One as-yet-unexplored facet of offloading is the extent to which offloaded information may serve as a retrieval cue for non-offloaded information. Imagine going to lunch with a friend who reminds you that the last time you met her at this restaurant, she ordered a cobb salad, so she plans to choose something different for lunch today. Your friend’s reminder about her previous lunch selection could also serve as a reminder for you of your own meal at that lunch outing: A grilled chicken sandwich. Similarly, imagine programming an event into your electronic calendar to remind you to attend an appointment with Dr. Smith. The appointment event may not include Dr. Smith’s office phone number, office address, or even the reason for the appointment. Instead, the “Dr. Smith” event description is thought to be sufficient to remind you of these related, but not directly offloaded, details.

In the present study, we were interested in how offloaded information may cue non-offloaded information using another type of knowledge that may be offloaded in everyday life: Information about medications (see Hargis & Castel, 2018a). Up to 80% of health-related information is forgotten immediately (Kessels, 2003), with nearly half of remembered medical information being remembered incorrectly (Anderson et al., 1979). Importantly, the loss of this information from memory can have significant downstream health consequences (Gellad et al., 2011; Hughes, 2004; Roebuck et al., 2011; see Hargis & Castel, 2018b for a review). Given the body of work described above suggesting that offloading can improve performance on memory-based tasks, it is possible that offloading medication information could benefit participants’ ability to preserve access to it. However, this strategy is not likely to be implemented by copying the doctor’s directions verbatim due to both the mismatch between writing speed and speech rate as well as the cognitive resources required to support effective note-taking (Piolat et al., 2005); instead, a patient who is offloading health-related information may be more likely to paraphrase or take note of key terms. To mimic this scenario, in the series of studies reported here, participants were provided the ability to choose to offload some, but not all, of the information presented to them. The use of a partial offloading paradigm allowed us to address a key question as to whether some approaches to partially offloading information served as a more effective retrieval cue than other approaches to partial offloading. We also further explored the extent to which people choose to engage in partial offloading in ways that optimize subsequent memory performance. Moreover, we examined whether people make similar choices about what to offload when information value (manipulated via severity of the health outcome, e.g., cough versus stroke; Hargis & Castel, 2018a) varied—compared to when it was held constant—to mimic real-world scenarios in which people may choose to offload health-related information.

## Rationale and the current research

In the current work, we aimed to address two important questions related to cognitive offloading for ecologically valid materials. First, we address the potential for partially offloaded information to support retrieval of related information that was not directly offloaded by comparing memory performance in a run of partial offloading trials (hereafter referred to as a block) to a run of internal memory trials (i.e., the internal memory block). We also tested which configuration(s) of partial information chosen for offloading provide the best support for retrieval of non-offloaded information by examining whether performance in the partial offloading block differs as a function of the missing information to be retrieved from memory. Understanding if and how partially offloaded information could cue retrieval of related information would provide important insights into the potential for offloading to support the performance of everyday tasks even when that information is not directly contained in the offloaded store. Finally, we turn to the relationship between offloading and importance. In our everyday lives, we encounter some information that is very important to remember (such as an upcoming doctor’s appointment) and some information that is relatively less important (such as an upcoming seminar at the local library). In light of the relative importance of these two events, participants may choose to partially offload information about the important event (e.g., the doctor’s appointment) and may not choose the less important event for offloading (e.g., library seminar; see Gilbert et al., 2020 for a similar pattern of value-based intention offloading; and see Park et al., 2022 for a similar pattern of value-based word list offloading). Additionally, participants may be able to rely on internal memory to retrieve information about the important event and may be less likely to encode details of the less important event into long-term memory; however, there is some evidence to suggest that choosing high-value items for intention offloading can serve to free up internal memory resources to be allocated toward remembering low-value information (Gilbert et al., 2020). Importantly, this prior work also demonstrated that if the external store becomes unavailable, only low-value information is retained in internal memory (Gilbert et al., 2020). We therefore extend this prior work by using ecologically valid memoranda and by testing the consequences for internal memory when only partial offloading is permitted in a retrospective memory task.

## Experiment 1: Memory for medication interactions

In Experiment 1, we conducted a within-subjects study testing memory for medication interactions. Memory for the health outcome, or memory for one of the two medications which resulted in that specific interaction outcome, was tested. For example, participants in this study may have seen “Norvasc + Antivert = bloating” at encoding. At the test, they could have been presented with “Norvasc + _____ = bloating” and were probed to retrieve, from memory, the medication that resulted in that health outcome when it interacted with the given medication (i.e., “Norvasc”). Alternatively, the participant may have been tested on their memory for the health outcome that resulted from the interaction of these two medications, as in “Norvasc + Antivert = _____”. Importantly, for the former test item (i.e., “Norvasc + _____ = bloating”) there were an equal number of trials for which memory for the first and second medications in each interaction was probed.

### Method

#### Participants

Young adult participants were recruited from the Stony Brook University Psychology Department participant pool to complete this asynchronous online study session lasting approximately 30 min. Recruited participants were targeted to be between the ages of 17 and 30 years and were awarded course credit for their participation at a rate of 1 credit per hour.

#### Procedure

After consenting to the study, participants were instructed that they would be shown a number of medication combinations and the health outcome that results from consuming those two medications together (e.g., a medication interaction), and that their memory for this information would be tested later. For example, participants might have seen “Macrobid + Synthroid = itching.” Medication stimuli were taken from a previously normed database (see Hargis & Castel, 2018a); medications were not highly familiar to participants (*M* = 2.29, SD = 0.42; on a scale from 1 being not at all familiar to 5 being very familiar). Health outcomes were normed prior to the initiation of these experiments using a separate sample of participants from Amazon’s Mechanical Turk (MTurk). Health outcomes included in this experiment were rated as mildly concerning (*M* = 2.71, SD = 0.16; on a scale from 1 being not at all concerning to 5 being very concerning); see Appendix for a complete list of medications and health outcomes for all Experiments.

Participants completed three (3) study-test cycles (hereafter referred to as blocks) and the same study information was displayed in each block, such as “Norvasc + Antivert = bloating.” Stimuli were displayed to participants for 7 s each during encoding (as in Hargis & Castel, 2018a). Each stimulus was unique in terms of the medications and the associated health outcome. Each block contained 18 to-be-remembered stimuli; all items were tested at the end of each block. Due to the asynchronous online nature of data collection for this study, participants also completed 3 catch trials per block were also included to ensure that present at their computers and were attending to the stimuli presented. Catch trials were presented for 7 s and consisted of simple instructions for participants to follow on that trial, such as “Please type the letter ‘f’.” Memory for non-catch trials was tested once for each piece of missing information; order was randomized across blocks (e.g., a participant might see “Norvasc + Antivert = _____” in Block 1, “Norvasc + ______ = bloating” in Block 2, and “______ + Antivert = bloating” in Block 3). For each memory probe, participants chose a response from a drop-down menu; a response was required for each trial before moving on to the next probe item. Item probes were equally distributed across position 1 missing, position 2 missing, and health outcome missing within each block, resulting in 6 probes per item type in each block (with each piece of missing information in each equation probed across the experiment).

### Hypotheses

We expected that participants’ performance would improve across the three blocks. We also expected that there would be differences in performance as a function of the missing information (position 1 versus position 2 versus health outcome), but we did not have a directional hypothesis regarding the nature of these differences.

### Statistical approach

We first tested whether performance on trials for missing medication information varied as a function of position using a paired-samples *t* test; if not, these items were collapsed together in subsequent analyses. Based on the outcome of this result, we planned to conduct a 3 (block: 1, 2, 3) × 2 (missing information: medication, health outcome) or 3 (block: 1, 2, 3) × 3 (missing information: position 1, position 2, health outcome) within-subjects analysis of variance (ANOVA). Follow-up contrasts were then conducted as necessary, using a Bonferroni correction to account for multiple comparisons.

### Power analysis and stopping rule

For this initial experiment, an a priori power analysis for the 3 × 3 within-subjects ANOVA suggested full, usable datasets from 48 participants; this sample size was expected to give us 85% power to detect a small-to-medium effect of 0.20 assuming a correlation of 0.50 among the repeated measures. Participants who took > 2 SDs from the mean completion time to complete the study were excluded, as were participants whose overall accuracy was > 2.5 SDs below the mean, or who failed to provide at least one correct answer per block. Participants who displayed average response times longer than 60 s in the test phase were also excluded. Participants who navigated away from the experimental browser window for > 20% of the total experiment time were excluded, as were participants who failed to provide correct responses to at least 1 of the 3 catch trials in a given block. At the end of the study, participants were asked if they were doing anything else during the experiment; those who reported doing so were excluded from the final dataset. Additionally, participants who reported that they experienced issues while completing the experiment that were more serious than “minor bumps” or who reported that they experienced any problems with the experimental program were excluded. Participants were also asked to report if they used any aides outside of those provided in the experiment to help them recall information over the course of the experiment, such as writing information down, taking screenshots of study trials, and the like. Participants who reported creating and using additional external aids to complete this experiment were excluded from further analysis. Additionally, participants who self-reported having previously participated in another study using these materials were excluded.

### Results

Usable data from 48 participants were collected asynchronously through an online experiment interface. Participants were from Stony Brook University’s Psychology Department participant pool. The final sample was 33.33% male, with an average age of 18.92 years (SD = 1.63; range 17–23 years). Twenty-six participants in the final sample identified as Asian/Pacific Islander, 14 participants identified as White, 3 participants identified as Other, 3 participants identified as Black, and 2 participants identified as Hispanic/Latinx.

To test our first research question (i.e., whether memory performance for medications differed as a function of position), a paired-samples* t* test was conducted. This test revealed no difference between memory for medication information in the first or second position,* t* (47) = − 1.187, 95% CI [− 0.047, 0.012], *p* = 0.241, suggesting that memorability of medication information is not related to presentation position.

Following this, we conducted a 3 (block: 1, 2, 3) × 2 (missing information: medication, health outcome) within-subjects ANOVA on task performance. We observed a main effect of block, *F* (2, 94) = 40.017, *p* < 0.001, as well as a nonsignificant main effect for missing information type, *F* (1, 47) = 1.460, *p* = 0.233, and a nonsignificant interaction between missing information type and block, *F* (2, 94) = 0.635, *p* = 0.532. As expected, participants’ performance improved across blocks (block 1 versus block 2: *t* (47) = 6.045, *p* < 0.001, block 1 versus block 3: *t* (47) = 8.393, *p* < 0.001, block 2 versus block 3: *t* (47) = 4.309, *p* < 0.001; all *p*-values survived Bonferroni correction). Importantly, memory performance was well above chance levels (where here chance would be 8.33%, both for medication information and interaction). However, contrary to our predictions, memory performance did not differ as a function of item probe type (medication, interaction).

### Discussion

The results of this experiment revealed no differences in memory for medication information as a function of position, nor were there differences in memory observed for medication information and interaction (i.e., health outcome) information. However, in accordance with our prediction, participants’ performance improved across blocks. These results suggest that memory for missing health information may be more idiosyncratic than originally predicted. Based on this finding, exploratory analyses will be undertaken in subsequent experiments to examine to what extent internal memory performance may relate to later offloading choice.

## Experiment 2: Partial offloading

In Experiment 1, memory for medication information and health outcomes was tested. Experiment 1 allowed us to calibrate the paradigm and motivated the hypothesis of Experiment 2 regarding the type of missing information (i.e., medications versus health outcome) having no specific impact on memory performance at test, thereby suggesting that benefits of offloading can be obtained regardless of what participants choose to offload. Here, we examined offloading choice behavior as a function of partial information that may be chosen for offloading. To return to the above example, the “Dr. Smith” event on one’s calendar may cue memory for the purpose of the appointment and the route to navigate to get to Dr. Smith’s office. While neither of these pieces of information is found directly in the calendar event, the provision of the Dr. Smith cue may be sufficient to bring these other related details to mind. In the current Experiment, we tested a similar scenario using medication interactions and outcomes in a condition under which participants were permitted to offload only two out of three relevant pieces of information in each trial.

### Method

#### Participants

A new set of young adult participants that did not participate in Experiment 1 were recruited from the Stony Brook University Department of Psychology participant pool to complete this asynchronously administered online experiment session lasting approximately 30 min. Recruited participants were targeted to be between the ages of 17 and 30 years. Participants were awarded course credit for their participation at a rate of 1 credit per hour.

#### Procedure

Participants were tested in three blocks: forced internal (standard memory for medication interactions condition), full offloading, and partial offloading blocks. The internal memory block was presented first for all participants, given that the choices participants make about offloading may be informed by their experience in the internal memory block. Following the initial internal memory block, the order of the offloading blocks (full, partial) was counterbalanced across participants. All other parameters, including stimulus timing, test parameters, and the like, were the same as those described in Experiment 1.

In the full offloading block, any trial chosen for offloading was saved in full (i.e., offloading the full item “Macrobic + Synthroid = itching”) and provided at test for reference. Critically, in the partial offloading block, participants were permitted to offload only two of the three relevant pieces of information. For example, a participant might have chosen to offload the two medications that are interacting but not the health outcome, or instead may have chosen to offload one medication and the health outcome resulting from the interaction with the non-offloaded medication. For both the full and partial offloading blocks, participants were permitted to save information on half of the trials in each block (9 trials total); non-offloaded trials were to be stored in internal memory. For up to 9 trials in the full offloading block, participants could choose to save the entire study item by clicking the “SAVE” button centered under the study item. If they did not want to save the study item, then they were instructed to click the “DON’T SAVE” button. On each trial of the partial offloading block, participants were given the opportunity to save up to 2 pieces of information per study item by clicking the “SAVE” button under each piece of information that they would like to offload. If they did not want to save any pieces of information from a study item, then they were instructed to click the “DON’T SAVE” button.

**Fig. 1:**
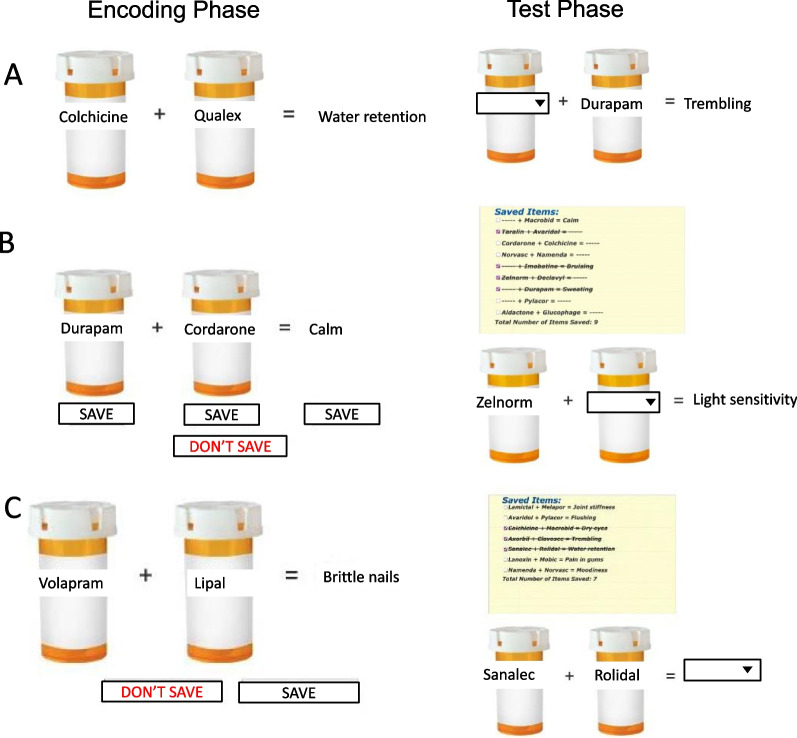
Task Schematic (Experiments 2 and 3) *Note.* Panel A depicts the internal memory block, panel B depicts the partial offloading block, and panel C depicts the full offloading block

Instructions provided to participants can be found in the Appendix. See also https://youtu.be/xZl220h-lTw for a video demonstration of the two offloading blocks, or visit https://psychology.psy.sunysb.edu/cam_lab/offloading_partial_DEMO_2021/ to run through an interactive demonstration; note that the first study block is a demonstration of the full offloading condition, and the second study block is a demonstration of the partial offloading condition. See Fig. 1 for a task schematic.

### Hypotheses

First, we expected that significantly higher accuracy would be observed in both the full offloading and partial offloading blocks compared to the forced internal block. Second, in the partial offloading block we expected that participants would be more likely to choose to offload the information type that resulted in the poorest performance in Experiment 1. Finally, we expected that choosing to offload the information type that resulted in the poorest performance in Experiment 1 would significantly benefit performance for the non-offloaded information within that trial in the partial offloading block.

### Statistical approach

#### Planned statistical analyses

We first tested for order effects (counterbalance order: internal memory, partial offloading, full offloading compared to internal memory, full offloading, partial offloading) to better understand whether subsequent analyses should control for counterbalance order.^1^

Our first hypothesis that performance would be better in the partial and full offloading blocks compared to the forced internal block was tested with a series of analyses of covariance (ANCOVAs) comparing (i) partial offloading to internal memory, and (ii) full offloading to internal memory, each controlling for counterbalance order. We prepared the data to test our second hypothesis (i.e., that for offloaded trials in the partial offloading block participants would be more likely to choose to offload the information type shown to be associated with the poorest performance in Experiment 1) by first classifying each piece of information in each trial (medication position 1, medication position 2, health outcome) as either being chosen for offloading or not chosen for offloading. Then, these values were summed across all participants and were submitted to a 2 (offloaded, not offloaded) × 3 (medication position 1, medication position 2, health outcome) chi-square test to examine any differences that may emerge in the choice to offload by information type. To test our final prediction (i.e., that the choice to offload the information type shown in Experiment 1 to be associated with the poorest performance would significantly benefit performance for the non-offloaded information), we conducted a *t-*test to compare performance on the offloaded trials only for the partial choice offloading types (medications only, medication (in either position) + health outcome) with performance for the non-offloaded piece of information as the dependent variable. This analysis was focused specifically on the subset of items for which participants (a) chose to offload information from that trial, and (b) were tested on the piece of information missing from the offloaded store. To illustrate, a trial for which a participant chose to offload “Norvasc + ____ = bloating” and was later tested for Medication 2 would be included in this analysis.

#### Planned exploratory questions and analyses

We conducted an exploratory analysis to test whether participants who performed more poorly on the forced internal block would choose to offload on a greater number of trials than participants who performed better on the internal memory block, as this remains an open question based on the prior literature (Morrison & Richmond, 2020; Risko & Dunn, 2015). We tested this question using a Spearman correlation, as these data violated the assumptions of normality necessary for a Pearson correlation.

Novel to our study, one important open question is the extent to which partial offloading benefitted performance compared to full offloading, particularly when participants were given the choice about what to offload. We did not have a strong a priori hypothesis regarding whether partial offloading would benefit performance similarly to full offloading on the whole, as the memorial benefits of offloading may be affected by the use of cognitive resources involved in the decision of what to offload. Instead, this effect was expected to depend on (i) the partial offloading type that is shown to confer larger benefits for performance, and (ii) the extent to which participants choose to partially offload this information type. Therefore, we conducted an exploratory analysis to test the central question regarding whether partial offloading benefitted performance to the same extent as full offloading by subtracting performance in the internal memory block from performance in the partial offloading block and the full offloading block, respectively, to create an “offloading benefit score” for each block. Offloading benefit scores (partial, full) were then submitted to an ANCOVA controlling for counterbalance order. We conducted a similar analysis for offloaded trials only to home in on the trials for which an offloading strategy was employed.

Finally, if we observed that participants showed variability in their offloading choices in the partial offloading block (e.g., choosing 2 of 3 elements to offloading sometimes, and only 1 of 3 elements for offloading on other trials), we planned to conduct a paired-samples *t*-test (or Wilcoxon signed-rank test as appropriate to our data) to test whether memory performance for non-offloaded information differed as a function of the number of elements offloaded.^2^ As a priori criteria for conducting this analysis were not met, it will not be discussed further.

### Power analysis and stopping rule

We powered the sample size for Experiment 2 to detect a difference between the benefit of full versus partial offloading with 85% power and a small-to-medium effect size of *d* = 0.35, which indicated a required sample size of 76 participants. Exclusion criteria described under Experiment 1 were applied to the current sample. Additionally, participants who did not choose to offload at least once in both the partial and full offloading blocks were not included in our final dataset, as well as participants who did not provide at least six offloaded trials with two elements in the partial offloading block. That is, a participant who chose to offload only one element—rather than two—more than three times was excluded from our final dataset. Further, in the partial offloading block, if a participant was not probed for the missing information for a partially offloaded item at least twice, their data were excluded from the analysis regarding the utility of partial offloading as cuing non-offloaded information (but were retained for other analyses of interest).

### Results

Usable data were collected from 76 participants from Stony Brook University’s Psychology Department participant pool. Data were collected asynchronously through an online experiment interface. The final sample was 36.84% male, with an average age of 19.12 years (SD = 1.37; range 17–23 years). Twenty-seven participants in the final sample identified as Asian/Pacific Islander, 32 participants identified as White, 2 participants identified as Other, 7 participants identified as Black, and 5 participants identified as Hispanic/Latinx. Three participants declined to report their racial/ethnic identity.

We first tested for order effects by conducting a 2 (block order: internal, full offloading, partial offloading versus internal, partial offloading, full offloading) × 3 (block type: internal versus full offloading versus partial offloading) repeated measures ANOVA to understand whether block order impacted performance. We observed a significant main effect of block type, *F* (2, 148) = 148.038,* p* < 0.001, but not of block order, *F* (1, 74) = 0.801,* p* = 0.374. The main effect of block type was qualified by a significant block type x block order interaction, *F* (2, 148) = 3.479,* p* = 0.003. Given the significant interaction observed here, subsequent analyses controlled for block order as appropriate.

**Fig. 2:**
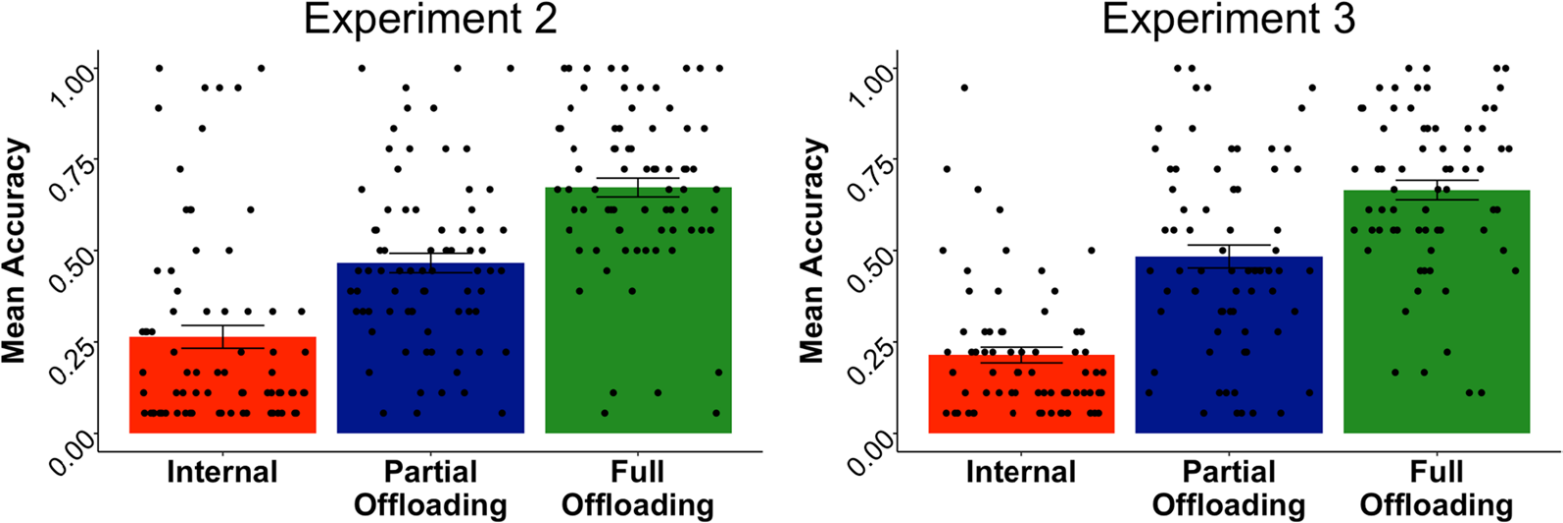
Accuracy by Block Type, Experiments 2 and 3

To test our first hypothesis, we conducted a series of two analyses of covariance (ANCOVAs) on block type (full offloading versus internal memory and partial offloading versus internal memory) controlling for block order. In both cases, we found that performance in the offloading blocks was significantly better than in the internal memory block (full offloading versus internal memory block: *F* (1, 75) = 202.803, *p* < 0.001; partial offloading versus internal memory:* F* (1, 75) = 77.684, *p* < 0.001; *M*_internal_ = 0.264, SD_internal_ = 0.272; *M*_full offloading_ = 0.672, SD_full offloading_ = 0.224; *M*_partial offloading_ = 0.466, SD_partial offloading_ = 0.231). These results support our hypotheses that participants would perform better in the offloading blocks compared to the internal memory block (see Fig. 2).

Contrary to our predictions for Experiment 1, we did not observe significant differences in performance by item type (medication, interaction). Based on this null result from Experiment 1, here we expected that performance would not differ as a function of the information type chosen for offloading. To test this hypothesis, we conducted a chi-square test on the information chosen for offloading in the partial offloading and observed no difference by information type$, \chi$^2^ (2) = 0.775, *p* = 0.678, providing support for our prediction of no difference based on what we observed in Experiment 1.

**Fig. 3:**
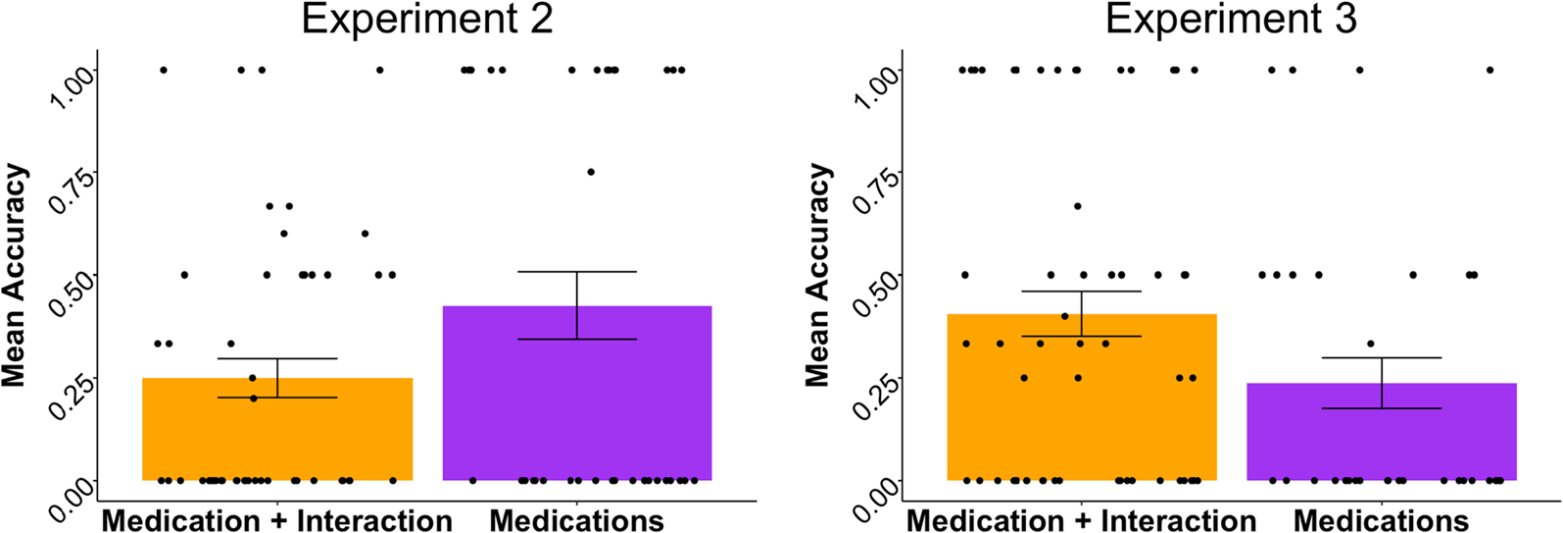
Internal Memory Performance in the Partial Offloading Block for Non-Offloaded Information, Experiments 2 and 3

Finally, to test whether performance in the partial offloading block differed as a function of the information chosen for offloading when the non-offloaded piece of information was tested (to explore the idea that some sorts of information may benefit memory for associated but not explicitly offloaded information more than others), we conducted a Welch’s *t*-test with approximate degrees of freedom and observed a marginally significant effect, *t* (59.191) = − 1.870, *p* = 0.067, with offloaded medication information only resulting in numerically better performance (*M*_med only_ = 0.426, SD_med only_ = 0.496) than the medication + interaction combination (*M*_med + interaction_ = 0.250, SD_med + interaction_ = 0.328). See Fig. 3.

### Exploratory analyses

We first wanted to examine whether participants with poorer internal memory chose to offload more, as findings on this point have been inconsistent in the extant literature (Morrison & Richmond, 2020; Risko & Dunn, 2015). Our data were found to violate the normality assumption necessary for a Pearson correlation according to the Shapiro–Wilk test for the number of trials saved (full offloading block: *W* = 0.361, *p* < 0.001; partial offloading block: *W* = 0.233, *p* < 0.001); therefore, Spearman correlations were conducted. Here, we observed no relationship between performance in the internal memory block and the number of trials on which participants chose to offload in the full offloading block, *rs* (74) = − 0.153, *p* = 0.188, nor in the partial offloading block, *rs* (74) = − 0.060, *p* = 0.605.

To test our second exploratory question regarding the magnitude of the benefit following full versus partial offloading, we conducted an ANCOVA on the offloading benefit scores (partial or full offloading block performance—internal memory block performance) controlling for block order. Here, we see that participants receive a bigger benefit to performance in the full offloading block (*M*_full offloading benefit_ = 0.410, SD_full offloading benefit_ = 0.250) compared to the partial offloading (*M*_partial offloading benefit_ = 0.202, SD_partial offloading benefit_ = 0.200) block, *F* (1, 75) = 106.971, *p* < 0.001. A similar pattern of benefit in the offloading blocks was observed when we restricted this analysis to only consider trials chosen for offloading in the full and partial offloading blocks (*M*_full offloading benefit_ = 0.649, SD_full offloading benefit_ = 0.320; *M*_partial offloading benefit_ = 0.333, SD_partial offloading benefit_ = 0.276), *F* (1, 75) = 198.297, *p* < 0.001.

### Discussion

We observed support for our hypothesis that offloading would benefit performance compared to internal memory alone, with both full and partial offloading conditions leading to better performance than relying on internal memory. We also found a pattern of results consistent with our prediction that performance would not significantly differ as a function of the information type chosen for offloading in the partial offloading condition, though we note that this interpretation is offered on the basis of a nonsignificant null hypothesis significance test. Future work examining similar questions may wish to adopt a Bayesian approach to data analysis to provide more information regarding whether this pattern is truly indicative of support for the null hypothesis (e.g., no difference in performance based on offloaded information). We also observed a numerical advantage for performance on the non-offloaded information when only medications were offloaded compared to offloading one medication and the interaction, but this difference failed to reach the threshold for statistical significance.

Our exploratory analyses revealed that there was not a significant relationship between performance in the internal memory block and the number of trials for which participants chose to offload in both the full and partial offloading blocks, consistent with prior work on offloading in retrospective memory (Brown, 2021; Burnett & Richmond, 2022; Morrison & Richmond, 2020). At the same time, we note that participants chose to offload nearly as often as they were allowed (e.g., participants offloaded 9 trials 99.4% of the time in the partial offloading condition and 96.3% of the time in the full offloading condition); therefore, this analysis suffered from a restricted range of behavior.

Perhaps most central to the current study, in comparing the performance benefits conferred by full versus partial offloading, we observe that full offloading benefits performance more than partial offloading. Together with our analysis showing that both offloading conditions benefit performance over internal memory, these results suggest that partial offloading may result in better performance than would be achieved by using internal memory alone but is not as helpful to performance as full offloading. We return to this point in detail in the General Discussion.

## Experiment 3: Partial offloading and medication interaction outcome importance

Participants may be drawn to offload information as a function of the subjective importance of the interaction outcome rather than by the anticipated difficulty of retaining information. In accordance with predictions following from the value-based remembering literature, we expected that the variation in interaction severity would provide participants with an inherent value-based structure for information importance that would serve to guide remembering. For example, Middlebrooks, et al. (2016) presented participants with potential allergens (e.g., peanuts, shellfish) that were assigned a value (0–10) to indicate how severe the reaction would be (low, medium, or high severity) if they were consumed by a person in the participant’s care, and found that both young and older participants used value-based mechanisms to remember the high-severity allergic reactions most accurately. Further to this point, Friedman and colleagues (2015) presented participants with medication interactions in three categories: mild (e.g., itching), moderate (e.g., heartburn), and severe (e.g., stroke). Participants remembered the severe items best (see Friedman et al., 2015, Experiment 1), indicating that participants consider vital health information to be worth knowing. Based on this prior work, we expected that the choice about what facets of information should be offloaded may vary across low-, medium-, and high-importance outcomes. To replicate and extend Experiment 2, health outcome importance was varied under the present Experiment in order to better understand how offloading choice behavior might change as a function of interaction outcome severity. All other methodological details are the same as outlined in Experiment 2.

### Method

#### Participants

A new set of young adult participants that did not participate in Experiments 1 or 2 were recruited from the Stony Brook University Department of Psychology participant pool to complete this asynchronously administered online experiment session lasting approximately 30 min. Recruited participants were targeted to be between the ages of 17 and 30 years and were awarded course credit for their participation at a rate of 1 credit per hour.

#### Procedure

Participants were tested in three blocks: forced internal (standard memory for medication interactions condition), full offloading, and partial offloading blocks. All other parameters, including instructions, stimulus timing, test parameters, and the like, were the same as those described in Experiment 2, except for interaction severity (as determined by ratings provided by a separate group of participants, see Appendix). Here, items with low (i.e., dry mouth; *M*_concern_ = 2.51, SD_concern_ = 0.13), medium (i.e., backache, *M*_concern_ = 3.02, SD_concern_ = 0.02), and high (i.e., internal bleeding; *M*_concern_ = 3.90, SD_concern_ = 0.21) subjective severity were tested.

### Hypotheses

We expected that memory performance would be significantly better in both the full offloading and partial offloading blocks compared to the forced internal block. Second, we expected that participants would choose to offload high-severity interactions more often than low-severity interactions in the full offloading block. Third, in the partial offloading block, we expected that participants would be more likely to choose to offload trials that both (i) have severe interaction outcomes listed, and (ii) contain the information type that is associated with the poorest internal memory scores. Although under our second and third hypotheses we expected that participants would choose to offload high-severity outcomes more often than low-severity outcomes given their subjective value, the advantage in internal memory for more important health outcomes (see Friedman et al., 2015; Middlebrooks et al., 2016) raises the possibility that participants would show the opposite pattern, choosing to offload low-severity outcomes while storing high-severity outcomes in internal memory. Finally, we expected that choosing to offload the information type that resulted in the poorest performance when held in internal memory would significantly benefit the performance for the non-offloaded information within that pair in the partial choice block.

### Statistical approach

#### Planned statistical analyses

We again tested for counterbalance order effects as described in Experiment 2; and planned to add order as a covariate to the following analyses if an order effect was observed.^3^

We planned to test our first hypothesis that performance would be better in the partial and full offloading blocks compared to the forced internal block using two ANCOVAs comparing (i) partial offloading to internal memory, and (ii) full offloading to internal memory. To test our second hypothesis that participants would choose high-severity outcomes for offloading more often than low-severity outcomes, we conducted a 3 (severity: low, medium, high) × 2 (offloaded, not offloaded) chi-square test for trials in the full offloading block. Our third hypothesis that for offloaded trials in the partial offloading block participants would be more likely to choose to offload the information type associated with poorest internal memory performance, as well as trials for which outcomes are more severe, was tested with a 3 (outcome severity: low, medium, high) × 6 chi-square test (the chi-square will test for differences between the following categories: Medication 1-offloaded, Medication 2-offloaded, Outcome-offloaded, Medication 1-not offloaded, Medication 2-not offloaded, Outcome-not offloaded) using a similar approach for this test as described above in Experiment 2.^4^

To test our final prediction that partial offloading choice behavior would not differ, based on no differences in memorability for different pieces of information being observed in Experiment 1, we conducted a *t*-test comparing performance on the offloaded trials only for the partial choice offloading types (medications only, medication (in either position) + interaction) with performance for the non-offloaded piece of information as the dependent variable.

#### Planned exploratory questions and analyses

We again tested the open question as to whether participants who perform more poorly on the forced internal block would choose to offload on a greater number of trials than participants who perform better on the internal memory block (Morrison & Richmond, 2020; Risko & Dunn, 2015) using a Spearman correlation.

Novel to the current study, we tested the extent to which medication interaction severity may drive offloading behavior. Following predictions from the value-based remembering literature (see, for example, Castel, 2007), interaction outcome severity may be expected to provide some structure that could guide attention to and memory for more severe (i.e., important) outcomes. However, participants may continue to attend more strongly to the information that will be most difficult to remember regardless of interaction severity. Therefore, we do not have a strong directional hypothesis regarding whether participants will choose to offload information differently based on interaction severity. To address this central question, we planned to conduct an exploratory analysis regarding whether medication interaction severity drives offloading behavior more than item memorability on the partial offloading block only for outcome severity (low, medium, high), information offloaded (medications only, medication (in either position) + interaction) with performance on the non-offloaded information as the outcome.^5^

Further, if Experiment 2 had provided adequate data to test whether memory performance for non-offloaded information as a function of one or two elements being chosen for offloading and a significant difference were observed, we planned to conduct this exploratory analysis again under Experiment 3. As we did not have adequate data to address this exploratory analysis in Experiment 2, we did not pursue this analysis under Experiment 3.

### Power analysis and stopping rule

We calculated our sample size estimate for the exploratory 3 × 3 within-subjects ANOVA with 85% power assuming a small-to-medium effect (partial *η*^2^ = 0.04) and a correlation among repeated measures at 0.25. This indicated that we would need to obtain usable data from 69 participants. We collected usable datasets from 70 participants in order to maintain counterbalancing of block order across participants. Exclusion criteria were the same as those described for Experiment 2.

### Results

Usable data were collected from 70 participants from Stony Brook University’s Psychology Department participant pool. Data were again collected asynchronously through an online experiment interface. The final sample was 37.14% male (1.43% of participants in the current study identified their gender as other), with an average age of 19.89 years (SD = 1.31; range 18–23 years). Twenty-seven participants in the final sample identified as Asian/Pacific Islander, 33 participants identified as White, 2 participants identified as Other, 2 participants identified as Black, and 4 participants identified as Hispanic/Latinx. Two participants in this sample declined to report their racial/ethnic identity.

We again tested for order effects by conducting a 2 (block order: internal, full offloading, partial offloading versus internal, partial offloading, full offloading) × 3 (block type: internal versus full offloading versus partial offloading) to understand whether block order impacted performance. We observed a significant main effect of block type, *F* (2, 136) = 171.243,* p* < 0.001, but not of block order, *F* (1, 68) = 0.742,* p* = 0.392. The main effect of block type was qualified by a significant block type x block order interaction, *F* (2, 136) = 6.229,* p* = 0.003. Given the significant interaction observed here, subsequent analyses controlled for block order as appropriate.

To test our hypothesis that performance would be better in the partial and full offloading blocks compared to the forced internal block, we conducted a series of two ANCOVAs on block type (full offloading versus internal memory and partial offloading versus internal memory) controlling for block order for each respective analysis. Again, in both cases, we found that performance in the offloading blocks was significantly better than the internal memory block (full offloading versus internal memory block: *F* (1, 69) = 324.808, *p* < 0.001; partial offloading versus internal memory:* F* (1, 69) = 101.208, *p* < 0.001; *M*_internal_ = 0.214, SD_internal_ = 0.180; *M*_full offloading_ = 0.665, SD_full offloading_ = 0.222; *M*_partial offloading_ = 0.483, SD_partial offloading_ = 0.262). See Fig. 2. These results replicated the effects observed in Experiment 2 and lend further support to our hypothesis that participants would perform better in the offloading blocks compared to the internal memory block.

We failed to find support for our hypothesis that participants would choose high-severity outcomes for offloading more often than low-severity outcomes, results of a 3 (severity: low, medium, high) × 2 (offloaded, not offloaded) chi-square test for trials in the full offloading block, $\chi$^2^ (2) = 0.331, *p* = 0.848, with offloading rates being very similar across severity levels (high-severity offloading = 198; medium-severity offloading = 204; low-severity offloading = 206).

Contrary to our predictions for Experiment 1, we did not observe significant differences in performance by item type (medication, interaction). Based on this null result from Experiment 1, here we expected that performance would not differ as a function of the information type chosen for offloading. However, we expected that participants would be more likely to choose to offload trials for which outcomes were more severe. To test this hypothesis, we conducted a 3 × 6 chi-square test (outcome severity: low, medium, high x information type: Medication 1-offloaded, Medication 2-offloaded, Outcome-offloaded, Medication 1-not offloaded, Medication 2-not offloaded, Outcome-not offloaded). Overall, results of this analysis failed to support our novel hypothesis regarding outcome severity and were otherwise consistent with expectations regarding the information type chosen for offloading derived from Experiment 1, $\chi$^2^ (10) = 4.033, *p* = 0.946.

To test our final prediction that there would be similar performance levels across offloading combinations based on seeing no significant differences in internal memory performance by item type in Experiment 1, we conducted a Welch’s two-sample *t*-test with approximate degrees of freedom comparing performance on the offloaded trials only for the partial choice offloading combinations (medications only, medication (in either position) + interaction) with performance for the non-offloaded piece of information as the dependent variable. Here, we observed a significant difference in performance across offloading combinations, *t* (75.106) = 75.106, *p* = 0.044; *M*_medications only_ = 0.237, SD_medications only_ = 0.354, *M*_medication + health outcome_ = 0.405, SD_medication + health outcome_ = 0.409, suggesting that offloading one medication and an interaction resulted in better performance for the non-offloaded information (e.g., medication name) compared to offloading medication names and being probed for the associated health outcome. See Fig. 3.

### Exploratory analyses

We again tested if participants who performed more poorly on the forced internal block chose to offload on a greater number of trials than did participants who performed better on the internal memory block (Morrison & Richmond, 2020; Risko & Dunn, 2015) using a Spearman correlation, as data violated the assumptions necessary for a Pearson correlation, and observed nonsignificant correlations in both the full offloading block, *rs* (68) = 0.129, *p* = 0.286, and the partial offloading block, *rs* (68) = − 0.125, *p* = 0.302.

Novel to the current study, we planned to test the extent to which medication interaction severity drove offloading behavior by conducting a 3 × 2 within-subjects ANOVA on the partial offloading block only for outcome severity (low, medium, high) and information offloaded (medications only, medication (in either position) + health outcome). However, we found that not all participants in our dataset had all possible combinations of offloading type and severity level, leading to some cells that had missing data. Because ANOVAs are not able to handle missing data, we instead implemented a linear mixed model approach, which has been suggested as an appropriate method for analyzing datasets with missing data (Gabrio et al., 2022). Our linear mixed effects model predicted performance in the partial offloading block on trials where participants chose to offload and the non-offloaded piece of information was tested with the interaction of fixed effects of outcome severity and information offloading, and random effects representing a random slope for Block Order and a random intercept for Participant, where participant and condition are correlated using the lmerTest package in R (Kuznetsova et al., 2014). Therefore, our model was specified as follows: Acc Non-offloaded ~ Offload Combination * Severity + (1 + Counterbalance Order | Participant). Here, we observed no significant main effects or interactions (smallest *p* = 0.071), suggesting that neither offloading combination nor interaction severity, as well as the interactive effect of these two factors, predicted performance for non-offloaded information. We return to this result in the General Discussion.

### Discussion

We replicated our finding from Experiment 2 that performance in both offloading blocks was significantly better than in the internal memory block. Surprisingly, and contrary to our prediction, we did not observe that participants chose to offload high-severity outcomes more often than low-severity outcomes. Finally, while we expected that performance would not differ as a function of the information type chosen for offloading based on the results of Experiment 1, we did expect that participants would be more likely to choose to offload trials for which outcomes were more severe. We did not observe a significant effect for this analysis.

Interestingly, we observed that offloading one medication and an interaction resulted in better performance for the non-offloaded information (e.g., medication name) compared to offloading medication names and being probed for the associated interaction. Based on the results of Experiment 1, we did not expect to observe a difference by offloading choice. This significant result fails to support this hypothesis and did not replicate the results observed in Experiment 2. We return to this in the General Discussion.

Exploratory analyses regarding internal memory performance and offloading frequency replicated the nonsignificant relationships reported in Experiment 2 and in prior work (Brown, 2021; Burnett & Richmond, 2022; Morrison & Richmond, 2020), but again we observed a restricted range for this test with offloading rates at 98.4% and 96.5% in the partial and full offloading conditions, respectively. Study designs that allow for more variability in offloading behaviors, via the inclusion of a greater number of trials and/or varying task difficulty, may be better suited for examination of the relationship between offloading behaviors and internal memory.

Finally, we did not observe that the combination of offloaded elements, interaction severity, nor the interaction of offloading combination and interaction severity, predicted performance for non-offloaded information in the partial offloading block, suggesting that the relationship between the elements chosen for offloading and the impact of severity may be somewhat idiosyncratic. However, we had sparse data to address this question, so we are cautious of drawing firm conclusions on the basis of this test. Future work may design studies that provide balanced data to better address this question.

## General Discussion

The overarching goal of this series of Experiments was to examine the potential for part of the information in a set to improve memory performance for non-offloaded information. Experiment 1 provided evidence that there was no difference in memory performance for medication names by position, nor was there a difference in performance between probes testing for medication names or interactions. The lack of differences observed for medication position and medication versus interaction memory allowed us to turn our focus to examining the effect of partial and full offloading on performance in subsequent experiments. Experiment 2 revealed that performance was better in the offloading blocks compared to the block in which participants had to rely on the internal memory. Perhaps unsurprisingly, the benefit to performance was larger when participants were permitted to offload all information in a trial (e.g., full offloading) compared to when they were permitted to offload only some information (e.g., partial offloading). Further, participants did not preferentially offload medication information versus one medication + interaction, in line with the finding from Experiment 1 that performance across all probed elements (medication 1, medication 2, interaction) did not differ. We also did not observe a statistically significant difference between internal memory performance for non-offloaded information depending on offloading combination (medications versus medication + interaction), but numerical differences in Experiment 2 indicated better performance when participants chose to offload a medication and the interaction and were probed for the missing medication.

Experiment 3 results replicated some key patterns reported in Experiment 2, and extended Experiment 2 by examining differing levels of interaction severity. Given prior work with these materials on value-directed remembering (Hargis & Castel, 2018a) we expected that interaction severity may have an impact on offloading choice and benefit, as well as on internal memory performance for non-offloaded information, contrary to our findings. However, Hargis and Castel (2018a, Experiment 1) found impacts of severity on memory performance for older, but not younger, adults, so it is possible that our young adult sample was not as sensitive to the severity of interactions as an older adult sample may have been (though note that performance among their younger adult sample was quite high by the third block). In this way, our results from Experiment 3 were consistent with research on value-directed offloading with words assigned varying point values that suggested the impact of value on performance was reduced when participants expected to have access to their offloaded store (Park et al., 2022), as in this study. Critically, Park and colleagues (2022) forced participants to offload information in their task, whereas in the current study participants were given the choice of what to offload. Together, these studies suggest that offloading can impact performance in value-based memory tasks regardless of the offloading manipulation employed (e.g., forced, choice).

Briefly, we replicated the pattern that full and partial offloading conditions resulted in better task performance than relying on internal memory alone. However, we did not find support for our prediction that participants would choose to offload high-severity trials more often than low-severity trials in the full offloading block. Following this, and the results in Experiment 1 indicating no difference in internal memory for different information types, in the partial offloading condition we did not observe differences in offloading rates as a function of outcome severity and component information chosen for offloading.

Finally, we observed a difference in performance for non-offloaded information in the partial offloading condition such that medication + interaction offloading resulted in better memory for the missing medication information compared to offloading medications and being probed for the outcome. This result was surprising, given the lack of statistical difference observed in Experiment 2, coupled with the numerical trends from Experiment 2 indicating better performance when both medications were offloaded. Testing for interactive effects of offloading combination and interaction severity on performance for non-offloaded information in the partial offloading block, we did not observe significant predictive power of either combination, severity, nor the interaction of these two factors. While this result is in line with the lack of effects observed for the memorability of different pieces of information and for the severity on offloading behavior reported above, this was a surprising result from the standpoint of literature regarding the memorability of high-value information. If anything, we would have expected that people would better retain high-value information in internal memory compared to lower-value information, even if they also chose to offload this information at high rates. Together, these results suggest that the benefits of partial offloading, while present overall, may be more idiosyncratic than we initially predicted.

Accordingly, imagine a scenario where a person creates a note to remind themself to discuss a new pain symptom that they have been experiencing each morning over the last four weeks with their doctor. One person may create a note that simply reads “pain,” with the expectation that this would trigger them to recollect episodes of the pain occurring in the morning hours and to share this information with their doctor. Another individual may create a note that reads “morning” expecting that it will provide a cue of the memory for the symptom (pain) and to discuss it with their doctor at an upcoming appointment. Yet another person may expect the reminder of the upcoming appointment itself to cue memory to discuss the symptom and time of day the symptom occurs with their doctor. Each of these may very well be viable strategy to adopt in order to meet the end goal of sharing this information with a physician. However, the strategy that works best for one individual may not be the best strategy for cueing related information in other individuals. This is an interesting possibility that is worthy of future exploration in studies well-powered to examine individual differences.

Importantly, we did observe benefits for offloading, consistent with prior research (e.g., Gilbert, 2015; Morrison & Richmond, 2020; Risko & Dunn, 2015), and our results extend prior work by showing that offloading of partial information can confer benefits for performance. While this result makes intuitive sense, our study is the first to directly test this question. Further, we observed a significant benefit of partial offloading for recall of non-offloaded information, particularly when that information contained a medication name and the outcome, suggesting that the benefit afforded by partial offloading is not only due to the presence of relevant information in the offloaded store. That is, partial offloading of information may not only provide some relevant information in the offloaded store at a later test, but it can also act as a useful memory cue for retrieval of non-offloaded information. Although this result was not observed in Experiment 2, this exciting result provides a jumping-off point for further exploration of the individual differences in, and conditions under which, partial offloading may confer benefits for recall of non-offloaded information. We note here the small number of trials that were available to contribute to this analysis (i.e., only trials for which the element that was not chosen for offloading was probed at test were considered here) as one possible source of variation across Experiments, which may explain the divergent findings across studies. Future work designed to test this question more directly may be necessary to better characterize the benefit of offloaded cues on memory for non-offloaded information.

Nonetheless, the current results suggest that partial offloading is not only beneficial for the portion of the information that is offloaded, but that partially offloaded information can also act as a helpful cue for prompting recall of related, but not offloaded, information. This result, however, is in opposition to what would be predicted by the part-set cuing literature, where consistent impairments in recall of non-cued information in the presence of cues are observed (e.g., Slamecka, 1968). A critical difference between the current Experiments and those that comprise the part-set cuing literature is the way in which cues are selected. Here, participants themselves choose the information that will be presented later as a cue, and these cues served to improve performance for non-cued (i.e., non-offloaded) information, whereas in the part-set cuing literature, cues are typically chosen at random from the study list. The current results, therefore, suggest that offloaded information has more in common with the types of cues that have been shown to benefit memory performance for non-cued information (Ratcliff & McKoon, 1994; Smith, 1977; Tulving, 1974) than part-set cues (e.g., Slamecka, 1968).

Zooming out, the results of these Experiments provide empirical support for the benefit of offloading some, but not all, relevant information, a common offloading strategy in everyday life. Further, our results suggest that there may be some idiosyncratic factors that govern the partial information that one chooses to offload. One such factor may be metacognitive confidence, as this factor has been shown to dictate offloading behavior in other paradigms (e.g., Boldt & Gilbert, 2019), and may explain why we failed to observe predicted differences in offloading behavior for interaction severity in Experiment 3. Future work may focus on characterizing the role of person-specific factors, such as metacognitive confidence, on partial offloading behavior.

## Conclusion

This is the first study, to our knowledge, to examine partial offloading behavior and benefit. We observed consistent evidence across two Experiments that partial offloading conferred benefits for performance above and beyond reliance on only internal memory, as well as evidence that partially offloaded information could serve as an effective retrieval cue for non-offloaded information. These results provide empirical support for the benefit of cognitive offloading, even when only some information is offloaded.

## Data Availability

Data and analysis code are available on the Open Science Framework (OSF). An experimental program will be made available upon request. The link to the authors’ OSF page is as follows: https://osf.io/yb8zx/.

## Appendix

### Medication names and familiarity, as rated by a separate group of participants

**Table:**

Medication name	Familiarity (1 = not familiar at all, 5 = very familiar)
Mobic	1.72
Nazix	1.74
Namenda	1.75
Declavyl	1.85
Axorbil	1.86
Macrobid	1.87
Imobatine	1.87
Melapor	1.88
Sanalec	1.88
Colchicine	1.91
Qualex	1.91
Taralin	1.93
Scopolamine	1.95
Deltasone	1.97
Rolidal	2.00
Lipal	2.05
Pylacor	2.05
Zelnorm	2.08
Fioricet	2.08
Floragin	2.08
Antivert	2.10
Volapram	2.11
Lypamol	2.14
Aldactone	2.16
Promazatol	2.24
Clavosec	2.29
Lanoxin	2.31
Avaridol	2.33
Glucophage	2.37
Nardil	2.37
Cordarone	2.38
Durapam	2.45
Baclofen	2.50
Lamictal	2.50
Norvasc	2.62
Synthroid	2.63

### Health outcomes and level of concern (rated by a separate group of participants) of the stimuli used in Experiments 1, 2, and/or 3

**Table:**

Health outcomes	Level of concern (1 = not concerning at all, 5 = very concerning)	Which experiment(s)
Calm	2.32	1, 2, 3—low
Vivid dreams	2.41	1, 2, 3—low
Sweating	2.49	1, 2, 3—low
Brittle nails	2.59	1, 2, 3—low
Flushed cheeks	2.59	1, 2, 3—low
Dry mouth	2.66	1, 2, 3—low
Dry eyes	2.73	1, 2
Flushing	2.73	1, 2
Moodiness	2.73	1, 2
Water retention	2.79	1, 2
Bloating	2.80	1, 2
Bruising	2.82	1, 2
Trembling	2.99	1, 2, 3—medium
Backache	3.01	1, 2, 3—medium
Pain in gums	3.01	1, 2, 3—medium
Joint stiffness	3.03	1, 2, 3—medium
Light sensitivity	3.03	1, 2, 3—medium
Arm pain	3.04	1, 2, 3—medium
Irregular heartbeat	3.63	3—high
Chest pain	3.75	3—high
Bloody urine	3.86	3—high
Difficulty breathing	3.87	3—high
Internal bleeding	4.14	3—high
Heart attack	4.15	3- high

### Instructions provided to participants

#### Forced internal block

*Encoding* “In this experiment, you will learn about medication combinations and their interactions. A medication interaction occurs when one drug affects the activity of another drug when both are used together.

During this task, you will study several medication interaction items, and your memory for this information will be tested later on. For each interaction that you study, you will see two medications and the outcome that would occur if they were to be taken together (e.g., "sweaty palms"). Each outcome will only occur once—that is, only one pair of medications will result in sweaty palms.

Please remember as much information about the substances and their interactions as you can. Your memory will be tested for either the health outcome that is associated with each pair of medications or for either one of the medications. You will only be tested on one piece of information.”

*Testing “*Your memory will now be tested for either the health outcome that is associated with each pair of medications or for either one of the medications. You will only be tested on one piece of information. For example, you may be presented with one of the following during this test.

1. ''______________ + Medication B = side effect'' and asked to recall Medication A.
2. "Medication A + _________ = side effect" and will be asked to recall Medication B.
3. Medication A + Medication B = ________" and be asked to recall the side effect.

Please take as much time as you need.”

#### Partial offloading block

*Encoding* “Each interaction you will see is composed of three elements: Medication A, Medication B and a side effect. On each trial, you will have the opportunity to choose whether you would like to save two of the three elements of the interaction that is shown on the screen. You will be able to save 9 interactions. To save an element, please click on the “SAVE” button below that element. Whichever two elements of the interaction you saved will be accessible to you at a later time.

If you do not wish to save any elements of the interaction, please click “DON’T SAVE.” Each interaction will be displayed on the screen for 7 s.

Please remember as much information about the substances and their interactions as you can. Your memory will be tested for either the health outcome that is associated with each pair of medications or for either one of the medications. You will only be tested on one piece of information.”

*Testing* **“**Your memory will now be tested for either the health outcome that is associated with each pair of medications or for either one of the medications. You will only be tested on one piece of information. For example, you may be presented with one of the following during this test.

1. ''______________ + Medication B = side effect'' and asked to recall Medication A.
2. "Medication A + _________ = side effect" and will be asked to recall Medication B.
3. Medication A + Medication B = ________" and be asked to recall the side effect.

You may consult the list of interactions that you saved in the previous trial to help you on this test.

Please take as much time as you need.”

#### Full offloading block

*Encoding “*On each trial, you will have the opportunity to choose whether you would like to SAVE the interaction that is on the screen.

If you choose to SAVE the interaction, you will have access to the entire interaction at a later time. Please click either “SAVE” or “DON'T SAVE" on each trial to indicate your choice.

Please remember as much information about the substances and their interactions as you can. Your memory will be tested for either the health outcome that is associated with each pair of medications or for either one of the medications. You will only be tested on one piece of information.

When you're ready to begin the experiment, click “Next.”

*Testing “*Your memory will now be tested for either the health outcome that is associated with each pair of medications or for either one of the medications. You will only be tested on one piece of information. For example, you may be presented with one of the following during this test.

1. ''______________ + Medication B = side effect'' and asked to recall Medication A.
2. "Medication A + _________ = side effect" and will be asked to recall Medication B.
3. Medication A + Medication B = ________" and be asked to recall the side effect.

You may consult the list of interactions that you saved in the previous trial to help you on this test.

Please take as much time as you need.”

## Footnotes

1 As a significant block type (internal offloading, full offloading, partial offloading) x counterbalance order (internal memory, partial offloading, full offloading versus internal memory, full offloading, partial offloading) interaction was observed (reported in detail below), subsequent planned analyses included condition as a covariate where relevant.

2 To be included in this analysis, we required that a participant would have to provide at least two one-element partial offloading trials where one of the non-offloaded elements was probed (e.g., choosing Medication 1 for offloading, and either Medication 2 or outcome was probed) as well as two two-element offloading trials for which non-offloaded information was probed (e.g., offloading Medication 1 and Medication 2 and the outcome is probed at test). We required that at least 50% of our participants (*n* = 38) provide data that meet the criteria outlined above to conduct this analysis. This criterion was not met, and therefore this exploratory analysis was not conducted.

3 Again, a significant block type (internal, full, partial) x counterbalance order (internal, full offloading, partial offloading versus internal, partial offloading, full offloading) interaction was observed, so we controlled for counterbalance order in subsequent analyses where appropriate.

4 To address the choice to offload different types of information more directly, if in the chi-square analysis differences in value were observed in the offloading cells by position (Medication 1-offloaded, Medication 2-offloaded, Outcome-offloaded), we planned to follow this analysis up by computing three bivariate Pearson correlations (or, alternatively, Spearman correlations should the data violate the assumptions of Pearson correlations) for scores in the internal memory block with the offloading rate in the partial offloading choice block by position (e.g., correlation 1: internal memory-Medication 1, offloading choice-Medication 1; correlation 2: internal memory-Medication 2, offloading choice-Medication 2; correlation 3: internal memory-Outcome, offloading choice-Outcome), and compare the correlation values to one another using the “cocor” R package (Diedenhofen & Musch, 2015). This effect was not observed, and therefore this follow-up analysis was not conducted.

5 Here, we originally planned to test this question with a 3 (interaction severity: low, medium, high) × 2 (information offloaded: medications only, medication + interaction) within-subjects ANOVA but sparse data precluded us from conducting this analysis as planned. Additional detail about the alternative analysis approach taken for this question can be found below.
